# Assigning new supergroups V and W to the *Wolbachia* diversity

**DOI:** 10.6026/97320630019336

**Published:** 2023-03-31

**Authors:** Amresh Kumar Sharma, Anup Som

**Affiliations:** 1 Centre of Bioinformatics, Institute of Interdisciplinary Studies, University of Allahabad, Prayagraj - 211002, India

**Keywords:** *Wolbachia*, Supergroups, 16S rRNA, Phylogeny, Average nucleotide identity (ANI), digital DNA-DNA hybridization (dDDH)

## Abstract

*Wolbachia* are endosymbiotic and alphaproteobacteria that belong to the order Rickettsiales. They are known to infect half of the insect population and cause host manipulation, and have been categorized into 19 monophyletic lineages called supergroups. Recently, two strains, wCfeJ and wCfeT were isolated from cat fleas (Ctenocephalides felis), but their supergroup relationships were not assigned. In this article, we have attempted to classify these two novel strains and establish their evolutionary lineage (i.e., supergroup designation). For this we performed 16S rRNA similarity analysis and reconstructed 16S rRNA phylogeny of 52 *Wolbachia* strains (including two novel strains) belong to 19 supergroups. We also performed average nucleotide identity (ANI) and digital DNA-DNA hybridization (dDDH) studies to measure genomic similarity between the two novel genomes. The results revealed that 16S rRNA similarity between the two novel strains is 97.94%, which is below the threshold value of 98.6% and phylogeny shows that they are placed at the two different positions (i.e., showing distinct evolutionary lineages). Further, genomic similarity analysis revealed that the novel genomes have ANI and dDDH values 79% and 22.4% respectively, which were below the threshold value of ANI (95%) and dDDH (70%). These results suggested that the novel strains neither shared a species boundary between them nor with any other previously identified supergroups, which designate them as two new supergroups, namely supergroup V (strain wCfeJ) and supergroup W (strain wCfeT).

## Background:

*Wolbachia* are alpha-proteobacteria that follows an endosymbiotic life and infect a wide range of arthropods and nematodes [1,2]. These bacteria are gram-negative, obligate and intracellular, and belong to the order Rickettsiales [3]. The genomes of *Wolbachia* have been analyzed to determine the type and nature of symbiotic relationshipsith their host [4, 5,6,7, 8]. Their nature of relationships in the hosts is reproductive parasite in arthropods, nutritional mutualists in bed bugs, and obligates mutualism in filarial nematodes [9, 10]. *Wolbachia* mediated all the reproductive manipulation in the host (i.e., mostly arthropods and some nematodes), by means of parthenogenesis, feminization, male-killing, by inducing cytoplasmic incompatibility, and nutritional supplement [11,12,13, 14,15]. It was estimated that *Wolbachia* infection is up to 40-76% of insects [16,17, 18]. *Wolbachia* have been classified into distinct monophyletic lineages called supergroups, which first came into the appearance in 1998 [19]. Later, Lo et al popularized this concept [20]. Till now, *Wolbachia* have been divided into 19 supergroups namely A-F, H-Q, S-U [21,22,[Bibr R23], 24]. The 16S rRNA gene is widely used in species determination and identification of new species or strains [25]. Comparison of 16S rRNA gene sequences allows differentiation/delineation of organisms at the genus, species and subspecies level. In the *Wolbachia* classification, the 16S rRNA gene played an important role in identifying and characterizing the new strains. In this paper, we focused on two recently published and undescribed *Wolbachia* genomes wCfeT and wCfeJ isolated from cat fleas (Ctenocephalides felis) found in co-infecting mechanism with the same host with different lifestyle such as strain wCfeJ is parasitic and wCfeT is mutualistic [26]. We used 16S rRNA genes to find out the evolutionary lineages of the novel strains in the *Wolbachia* diversity that currently have 19 supergroups. A total of 50 *Wolbachia* strains from the existing 19 supergroups and two novel strains were used in this study. Further, we did genomic similarity study on the genomes of the two novel strains using average nucleotide identity (ANI) and digital DNA-DNA hybridization (dDDH) test.

## Materials and methods:

## Data collection:

For finding the supergroup relationships of the two novel *Wolbachia* genomes wCfeT and wCfeJ, first, we took 16S rRNA sequences for phylogenetic analysis because it is highly conserved gene and able to show species delineation. 16S rRNA phylogeny is used to check whether two strains wCfeJ and wCfeT cluster with each other or with any other supergroup(s). For this, we used two novel strains plus other 50 strains from 19 supergroups which consist of a total of 52 *Wolbachia* strains in the study. All the 16S rRNA genes were downloaded from the NCBI database [27]. Details of the 52 *Wolbachia* strains are given in Table 1.

## Sequence similarity measure and Phylogenetic tree reconstruction:

16S rRNA sequence similarity paved a way for species demarcation among all bacterial species. So we firstly performed similarity check on the two novel strains along with other 50 strains using GGDC online server [28]. Further, we did phylogenetic analysis and reconstructed the 16S rRNA phylogeny. We aligned the sequences with CLUSTAL W package [29]. We also performed the model test by using ModelFinder that revealed HKY+F+I+G4 is the best suitable model [30]. Then maximum likelihood (ML) tree was reconstructed by using the IQTREE package with the HKY+F+I+G4 model [31].

## ANI measure and dDDH study:

After 16S rRNA similarity and phylogeny, we also measured the genomic similarity of the novel strains. For that, we performed average nucleotide identity (ANI) test and digital DNA-DNA hybridization (dDDH) study. ANI measures nucleotide-level genomic similarity between the coding regions of two genomes and here we attempted to find the divergence of the genomes to check whether two novel genomes are from the same supergroup or belong to different supergroups. We also carried out dDDH analysis to calculate *in-silico* genome-to-genome comparison using the GGDC tool [28]. The dDDH analysis emerged as an alternative to the tedious wet-lab DNA-DNA hybridization of species delineation. In GGDC tool, we used GBDP (Genome Blast Distance Phylogeny) method to calculate the probability that an inter-genomic distance yielded a dDDH value lower than 70 % considered as a novel species-delimitation threshold.

## Results and Discussion:

## Sequence similarity and phylogenetic analysis of 16S rRNA gene:

At first, we compared 16S rRNA sequence similarity of the two novel strains and found that similarity is 97.94 %, which is below the previously described threshold for species demarcation 98.6% [32, 33]. This result indicates that the novel strains were not from the same supergroup. Furthermore, we also analysed the sequence similarity of the novel strains with respect to the 50 other *Wolbachia* strains. Similarity results of the novel strains with other supergroups' starins showed that similarity score of the novel strains is lower than the threshold value of 98.6% (Table 2). Overall, 16S rRNA gene similarity result indicates that the novel strains neither belong to the same supergroup nor belong to any other supergroups.

**Figure 1:**
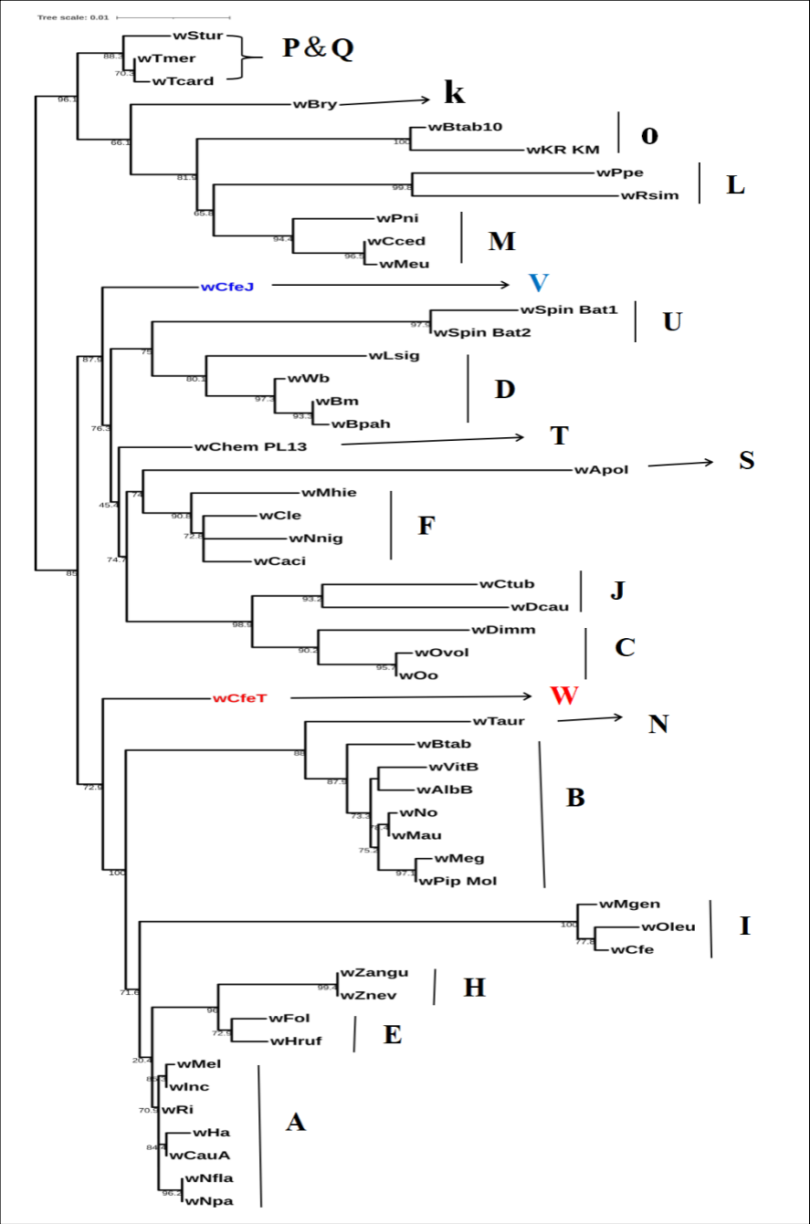
16S rRNA gene phylogeny of 52 *Wolbachia* strains with 19 supergroups showing position of two novel strains wCfeT (W) and wCfeJ (V). Maximum likelihood tree was reconstructed by using IQTREE with model HKY+F+I+G4. Supergroups are indicated against the strains.

Further, 16S rRNA ML phylogenetic tree was reconstructed using the HKY+F+I+G4 model of nucleotide evolution given in Figure 1. In this tree, two novel strains found in the different evolutionary lineages (i.e., not having a common ancestry) indicating that they did not belong to any other previously described supergroups. Here, we found that strain wCfeJ, shows parasitic nature with its hosts, is placed as an outgroup with supergroups C, D, F, J, S, T, and U. The bootstrap value at the node (87.9%) showing the reliability of the node. And strain wCfeT, shows mutualistic nature with its hosts, is placed as an outgroup with supergroups A, B, E, H, I, and N. The bootstrap value at this node is 72.9%, showing reliability of this node. In summary, 16S rRNA gene phylogeny confirms that strains wCfeJ and wCfeT having different lifestyle and has distinct evolutionary lineages.

## Genome comparison of novel strains:

The supergroups are sub-species level and their genomes are close enough to each other, so genomic divergence analysis is required for supergroup identification. Accordingly, we performed ANI analysis and dDDH study. We found that ANI and dDDH value between strains wCfeT and wCfeJ were found to be 79% and 22.4% respectively. The threshold value for ANI and dDDH are >95% and >70% respectively when the species belong to the same supergroup [32]. This result clearly indicates that the two novel strains are different. Further, the genomic contents of wCfeJ (NZ_CP051157.1) were 1.50Mb genome length; 1228 proteins; 35.2 GC%; 3 rRNA; 34 tRNA; 4 other RNAs; 1,463 genes and 194 pseudogenes. For the wCfeT (NZ_CP051156.1) were 1.20Mb genome length; 1070 proteins; 35.6 GC%; 3 rRNA; 34 tRNA 4 other RNAs; 1,155 genes and 44 pseudogenes. These genomic features show that the novel strains have genomic variations. The genomic analysis results also indicate that the novel strains do not share a species boundary.

## Conclusions:

The results of 16S rRNA based similarity analysis and phylogenetic study, and furthermore genomic ANI and dDDH analyses suggested that the novel strains neither shared a species boundary between them nor with any other previously identified supergroups, which designate them as two new supergroups, namely supergroup V (strain wCfeJ) and supergroup W (strain wCfeT). Therefore, our results aid new insights into the *Wolbachia* diversity and dynamics that will be useful in future comparative studies.

## Figures and Tables

**Table 1:** Details of 52 *Wolbachia* strains used in this study

**Host Species**	**Strain**	**Supergroups**	**Accession No.**
*Ctenocephalides felis*	wCfeT	Novel strain	*NZ_CP051156.1 (HF197_RS05045)
*Ctenocephalides felis*	wCfeJ	Novel strain	*NZ_CP051157.1 (HF196_RS00025)
*Drosophila melanogaster*	wMel	A	*NC_002978.6 (GQX67_RS05935)
*Nomada panzeri*	wNpa	A	*NZ_LYUX01000081.1 (BA050_RS05445)
*Nomada flava*	wNfla	A	*NZ_LYUW01000079.1 (BA052_RS05590)
*Drosophila simulans*	wHa	A	*NC_021089.1 (WHA_RS05510)
*Drosophila incompta*	wInc	A	*CP011148.1 (WG67_RS05300)
*Carposina sasakii*	wCauA	A	*CP041215.1 (wCauA_RS01020)
**Wolbachia* sp. (wRi)*	wRi	A	*(NC_012416.1 (WRI_RS06005)
*Drosophila simulans*	wNo	B	*NC_021084.1 (WNO_RS03275)
*Culex molestus*	wPip Mol	B	*NZ_CTEH01000001.1 (WPM_RS00430)
*Nasonia vitripennis*	wVitB	B	*NZ_GL883637.1 (WVB_RS0105690)
*Aedes albopictus*	wAlbB	B	*NZ_RWIK01000001.1 (EJE47_RS00350)
*Chrysomya megacephala*	wMeg	B	*CP021120.1 (CAI20_RS02875)
*Drosophila mauritiana*	wMau	B	*CP034335.1 (EJB00_RS01065)
*Bemisia tabaci*	wBtab	B	*CP016430.1 (BBB02_RS03415)
*Onchocerca ochengi*	wOo	C	AJ010276.1
*Onchocerca volvulus*	wOvol	C	AF069069.1
*Dirofilaria immitis*	wDimm	C	KU255236.1
*Brugia malayi*	wBm	D	*NC_006833.1 (WBM_RS02885)
*Wuchereria bancrofti*	wWb	D	*NZ_NJBR02000071.1 (CCY16_RS03430)
*Brugia pahangi*	wBpah	D	*NZ_CP050521.1 (WBP_RS01130)
*Litomosoides sigmodontis*	wLsig	D	*CP046577 (GOY07_RS01740)
*Folsomia candida*	wFol	E	*NZ_CP015510.2 (ASM33_RS07175)
*Hypochthonius rufulus*	wHruf	E	MN699328.1
*Coptotermes acinaciformis*	wCaci	F	DQ837197.1
*Nasutitermes nigriceps*	wNnig	F	DQ837204.1
*Cimex lectularius*	wCle	F	*NZ_AP013028.1 (WCLE_RS01905)
*Madathamugadia hiepei*	wMhie	F	*NZ_WQMP01000029.1 (GO685_RS01815)
*Zootermopsi angusticollis*	wZangu	H	AY764279.1
*Zootermopsi nevadensis*	wZnev	H	AY764280.1
*Orchopeas leucopus*	wOleu	I	AY335924.1
*Ctenocephalides felis*	wCfe	I	AY157512.1
*Myodopsylla gentilis*	wMgen	I	AY335918.1
*Cruorifilaria tuberocauda*	wCtub	J	*CP046579 (GOY13_RS01470)
*Dipetalonema caudispina*	wDcau	J	*CP046580 (GOY14_RS02280)
*Bryobia sps*	wBry	K	EU499316.1
*Radopholus similis*	wRsim	L	EU833482.1
*Pratylenchus penetrans*	wPpe	L	*NZ_MJMG01000007.1 (BIY23_RS03360)
*Cinara cedri*	wCed	M	JN384079.1
*Macrosiphum euphorbiae*	wMeu	M	JN109113.1
*Pentalonia nigronervosa*	wPni	M	KJ786951
*Toxoptera auranti*	wTaur	N	JN384094.1
*Bemisia tabici*	wBt10	O	KF454771.1
*Kaburagia rhushicola*	wKM KR	O	MT554837.1
*Syringophilopsis turdus*	wStur	P	KP114103.1
*Torotrogla merulae*	wTmer	P	KP114102.1
*Torotrogla cardueli*	wTcard	Q	KP114101.1
*Atemnus politus*	wApol	S	*NZ_JAAXCS010000017.1 (HET73_RS02035)
*Cimex hemipterous*	wChem PL13	T	*NZ_CP061738.1 (ID128_RS02485)
*Spinturnix mites*	wSpin Bat1	U	KP165041
*Spinturnix mites*	wSpin Bat2	U	KP165042
*In cases where accession ID for the genes was not available, accession ID of the genome along with locus tag (in the parenthesis) of the gene has been mentioned

**Table 2:** 16S rRNA sequence similarity of the novel strains (wCfeT and wCfeJ) with respect to the 50 *Wolbachia* strains belong to 19 supergroups.

***Wolbachia* strain**	**Supergroup**	**16S rRNA similarity (in %)**	
		**wCfeT**	**wCfeJ**
wRi	A	98.37	98.47
wInc	A	98.1	98.4
wCauA	A	98.2	98.4
wMel	A	98.34	98.34
wNfla	A	98.47	98.27
wNpa	A	98.47	98.27
wHa	A	98.4	98.2
wPip Mol	B	97.07	97.14
wMau	B	97.07	97.01
wNo	B	97.01	96.94
wAlbB	B	97.27	97.2
wMeg	B	96.94	97.01
wBtab	B	96.87	96.94
wVitB	B	96.74	96.67
wOo	C	96.47	96.47
wOvol	C	96.34	96.47
wDimm	C	96.34	97.07
wBm	D	97.79	97.8
wWb	D	97.6	97.94
wBpah	D	97.34	97.8
wLsig	D	97.27	97.54
wHruf	E	98.38	97.94
wFol	E	98.2	98.01
wMhie	F	97.54	97.87
wCle	F	97.94	98.2
wNnig	F	97.81	97.81
wCaci	F	98.1	98.1
wZangu	H	98.16	98.28
wZnev	H	98.16	98.28
wCfe	I	95.76	95.76
wMgen	I	95.7	95.77
wOleu	I	95.46	95.69
wDcau	J	95.94	96.54
wCtub	J	96.54	97.07
wBry	K	97.47	97.26
wRsim	L	95.97	95.38
wPpe	L	95.93	95.41
wMeu	M	97.78	97.59
wPni	M	97.07	96.94
wCced	M	97.72	97.8
wTaur	N	96.63	96.44
wKR KM	O	96.96	96.77
wBtab10	O	96.65	96.37
wTmer	P	98.23	98.23
wStur	P	97.88	97.88
wTcard	Q	97.92	98
wApol	S	95.5	95.91
wChem PL13	T	98.07	98.47
wSpin Bat1	U	97.69	98.27
wSpin Bat2	U	97.41	97.98

## References

[1] Sironi M (1995). Molecular and Biochemical Parasitology.

[2] Werren JH (1995). Proc R Soc Lond B.

[3] Harris HL (2010). Symbiosis.

[4] Hoerauf A (2003). Medical Microbiology and Immunology.

[5] Duron O (2007). Heredity.

[6] Hosokawa T (2010). Proc Natl Acad Sci USA.

[7] Lindsey ARI (2016). Genes Genomes Genetics.

[8] Badawi M (2018). Genes.

[9] Bouchon D (1998). Proc R Soc Lond B.

[10] Kageyama D (2002). Heredity.

[11] Werren JH (2008). Nat Rev Microbiol.

[12] Cordaux R (2011). Trends in Genetics.

[13] Miyata M (2017). Biol Lett.

[14] Beckmann JF (2017). Nat Microbiol.

[15] Perlmutter JI (2019). PLoS Pathog.

[16] Hilgenboecker K (2008). FEMS Microbiology Letters.

[17] Zug R (2012). PLoS ONE.

[18] Kajtoch L (2018). Peer J.

[19] Zhou W (1998). Proc Biol Sci.

[20] Lo N (2002). Molecular Biology and Evolution.

[21] Lefoulon E (2020). BMC Microbiol.

[22] Laidoudi Y (2020). IJMS.

[R23] Konecka E (2021). Infection, Genetics and Evolution.

[24] Olanratmanee P (2021). Southeast Asian J Trop Med Public Health.

[25] Hassler HB (2022). Microbiome.

[26] Driscoll TP (2020). Peer J.

[27] https://www.ncbi.nlm.nih.gov/.

[28] Meier-Kolthoff JP (2013). BMC Bioinformatics.

[29] Thompson JD (1994). Nucleic Acids Res.

[30] Kalyaanamoorthy S (2017). Nat Methods.

[31] Trifinopoulos J (2016). Nucleic Acids Res.

[32] Kim M (2014). International Journal of Systematic and Evolutionary Microbiology.

[33] Caudill MT (2022). Microorganisms.

